# Establishment of a neonatal rat model of sequential hyperoxic hypoxia to recapitulate clinical progression of bronchopulmonary dysplasia-associated pulmonary hypertension

**DOI:** 10.1186/s40635-025-00822-z

**Published:** 2025-11-04

**Authors:** Dan Wang, Siqi Hu, Jingke Cao, Haoqin Fan, Ye Ma, Fan Yang, Changgen Liu, Shanghong Tang, Zhichun Feng, Yunbin Xiao, Qiuping Li

**Affiliations:** 1 Department of Cardiology, The Affiliated Children’s Hospital of Xiangya School of Medicine, Central South University (Hunan Children’s Hospital), Changsha, China; 2 Department of Neonatology, Department of Newborn Care Center, Senior Department of Pediatrics, The Seventh Medical Center of PLA General Hospital, Beijing, China; 3 Institute of Pediatrics, Senior Department of Pediatrics, The Seventh Medical Center of PLA General Hospital, Beijing, China; 4 Pediatric Intensive Care Unit, Shenzhen Baoan Women’s and Children’s Hospital, Shenzhen, China; 5 The Second School of Clinical Medicine, Southern Medical University, Guangzhou, China

## Abstract

**Background:**

Bronchopulmonary dysplasia-associated pulmonary hypertension (BPD-PH) seriously threatens the lives of preterm infants. The absence of animal models that can simulate its progression from early hyperoxic lung injury to late hypoxic vascular remodeling has hindered related research.

**Objective:**

To establish a neonatal rat BPD-PH model by simulating exposure to sequential hyperoxic hypoxia experienced by human preterm infants.

**Methods:**

Newborn SD rats were randomized into two control groups (C1 exposed to 21% O₂ for 2 weeks; C2 exposed to 21% O₂ for 3 weeks), and three exposure groups (H1 exposed to 75% O₂ for 2 weeks; H2 exposed to 75% O₂ for 2 weeks and then to 10% O₂ for a week; H3 exposed to 75% O₂ for 2 weeks and then to normoxia for a week). Cardiopulmonary parameters were evaluated by echocardiography, right ventricular systolic pressure measurement, histology, and α-SMA immunofluorescence.

**Results:**

H1 and H2 groups exhibited distinct phenotypes, with those in the H2 group showing more severe phenotypes. The H2 group exhibited a 142% increase in RVSP relative to those in the C2 group. The right-heart index (RI) was 0.43 ± 0.01 in the H2 group, 0.36 ± 0.02 in the H3 group, and 0.22 ± 0.03 in the C2 group. Pulmonary vascular remodeling was significantly increased in the H2 group compared to the control and H3 groups. The H2 group uniquely replicated the disease process, with alveolar simplification preceding hypoxia-induced vascular thickening.

**Conclusion:**

The sequential hyperoxic hypoxia model dynamically mimicked the clinical progression of BPD-PH, which may provide a powerful platform for stage-specific mechanism research and development of novel therapeutic strategies.

## Background

Premature birth remains a global health burden, with extremely preterm infants (< 28 gestational weeks) at risk for bronchopulmonary dysplasia (BPD) and BPD-associated pulmonary hypertension (BPD-PH) [1, 2]. Up to 60% of extremely low birth weight infants develop BPD [3], and 25–43% of severe cases progress to BPD-PH, with mortality rates exceeding 30% [4]. BPD-PH presents with heterogeneous clinical phenotypes, including a significant patient subset that experiences early hyperoxic injury followed by chronic hypoxic episodes after discharge due to persistent desaturations, limited home oxygen access, or gradual oxygen weaning [5, 6]. The pathogenesis involves hyperoxia-induced oxidative stress disrupting alveolarization, while subsequent hypoxic conditions in these specific patients promote pulmonary vascular remodeling and right ventricular dysfunction [7]. Despite this biphasic trajectory in clinically relevant patient subgroups, the causal link between early hyperoxic injury and subsequent hypoxic vascular pathology remains poorly defined [8].

Animal models are essential for mechanistic studies [9], but existing BPD-PH models inadequately replicate this specific disease progression pattern. Prolonged hyperoxia models (60–100% O₂, 14 days) induce alveolar simplification but inconsistently generate pulmonary hypertension, with high mortality rates [10, 11]. The threshold of 75% O₂ was chosen based on previous studies demonstrating significant but reproducible injury at this concentration. A higher oxygen concentration results in a higher mortality rate during model establishment, without significantly improving the success rate [12, 13]. Hypoxia-only models fail to mimic the primed inflammatory microenvironment of early BPD [14]. Critically, no model replicates the sequential hyperoxia-to-hypoxia transition observed in this clinically important patient subset [15].

While acknowledging that many BPD patients receive continuous oxygen supplementation, a substantial subgroup experiences different clinical trajectories involving periods of chronic hypoxia. This hypoxic phase drives smooth muscle proliferation and endothelial dysfunction, yet management strategies for this patient subgroup lack mechanistic rationale [16]. It is therefore imperative to develop models that accurately mimic the disease progression in this clinically important subset [17].

Here, we present a novel sequential hyperoxia–hypoxia model replicating this specific BPD-PH phenotype in neonatal rats. Combining 14-day 75% O₂ exposure (early ventilator support) with 7-day 10% O₂ exposure (simulating chronic hypoxia in the aforementioned patient subgroup), this model integrates alveolar simplification, pulmonary vascular remodeling, and right ventricular hypertrophy into a comprehensive platform for investigating phenotype-specific therapies.

## Methods

### Animal hyperoxic exposure

The animal experimental protocols were approved by the Ethics Committee of the Hunan Children’s Hospital (HCHDWLL-2023-01). Timed pregnant SD (Sprague Dawley) rats (Hunan SJA Laboratory Animal Co., Ltd., Changsha, China) were housed under a 12-h light–dark cycle, with free access to standard food and water.

**Fig. 1:**
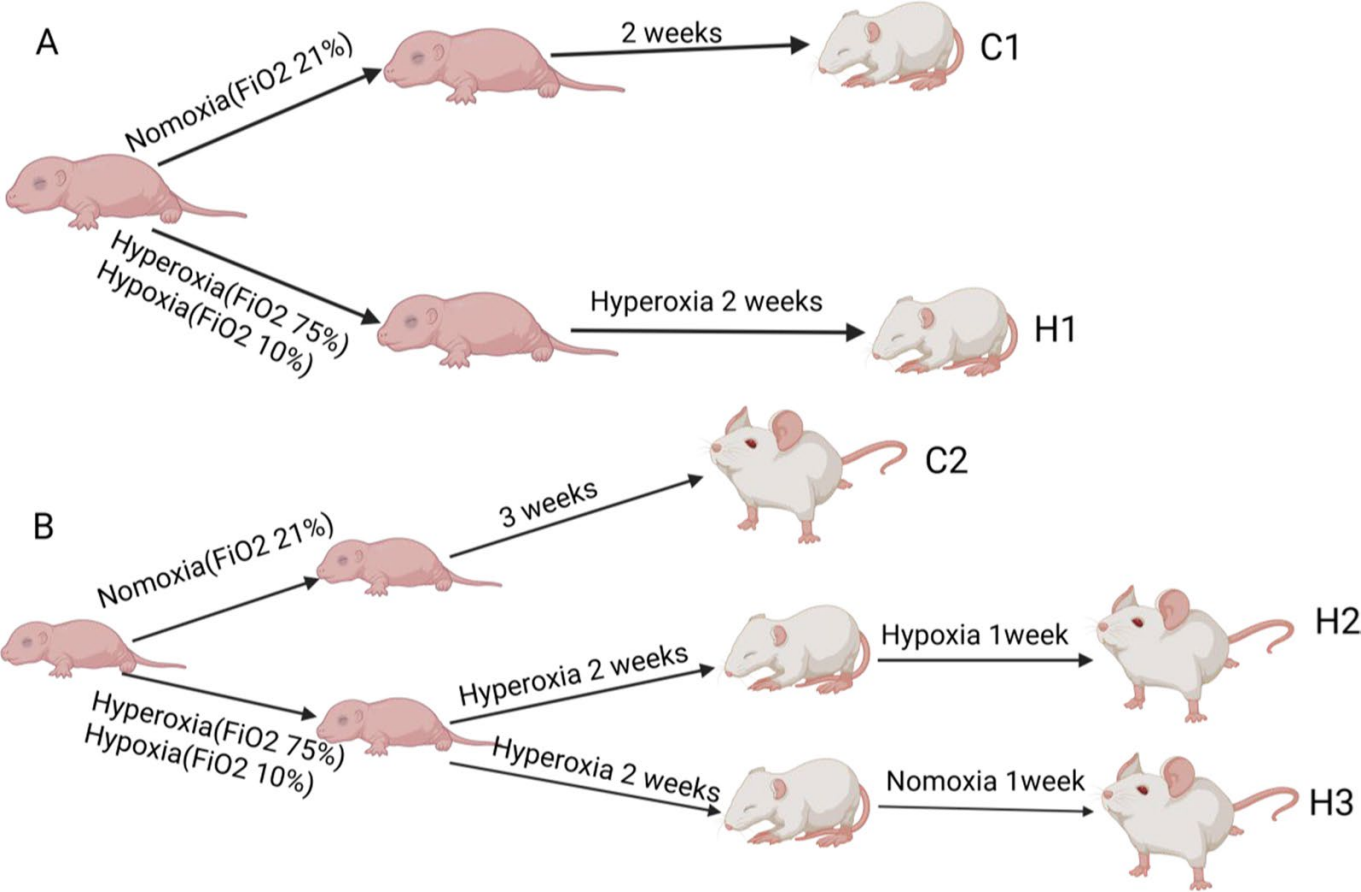
The procedures of the bronchopulmonary dysplasia-associated pulmonary hypertension model. C1 represents control group 1. C2 represents control group 2. H1 represents group 1. H2 represents group 2. H3 represents group 3

Within 24 h after birth, the litters were pooled and randomly assigned to either the control or exposure groups. Animals in the two control groups were maintained in a 21% O₂ environment for two and three weeks, respectively, and those in the exposure groups were divided into three subgroups, as shown in Fig. 1:

- Control groups:
  o C1: Normoxia for 2 weeks
  o C2: Normoxia for 3 weeks
- Exposure groups:
  o H1: 75% O₂ for 2 weeks
  o H2: 2-week 75% O₂ → 1-week 10% O₂
  o H3: 2-week 75% O₂ → 1-week normoxia

The procedures of model establishment are shown in Fig. 1A, B. Oxygen concentration was maintained at 75 ± 2% or 10 ± 2% using an automated gas controller. To prevent oxygen toxicity in maternal rats and maintain their lactation, the exposed groups were daily exchanged with maternal rats in normoxic conditions. Rats in each group were euthanized at the appropriate time for subsequent experiments.

### Echocardiography (ECG)

Rats were anesthetized with isoflurane and placed on a heating plate to keep their body temperature at 37 °C. Heart rates were monitored continuously and maintained above 300 bpm after inhalation induction. After shaving the precordial area, ECG was performed using a Vevo 2100 Imaging System equipped with an MS250 (21 MHz) probe (Philips). Right heart function parameters, including tricuspid annular plane systolic excursion (TAPSE), right ventricular area (RV), left ventricular area (LV), and the pulmonary acceleration time (PAT)/pulmonary ejection time (PET) ratio, were measured [18].

### RVSP measurement

Following ECG, right ventricular systolic pressure (RVSP) was measured using a terminal invasive hemodynamic examination via right ventricular catheterization. The anesthetized rat was fixed on a plank. The right jugular vein was isolated and intubated. A PE-50 tube filled with heparin saline was connected to a pressure sensor (Techman, Chengdu, China) and inserted into the right external jugular vein. A smooth ventricular pressure waveform indicated successful catheter placement in the right ventricle. RVSP was recorded, and RVSP was analyzed [19, 20].

### Histologic analysis

After hemodynamic measurement and sampling, lung and heart specimens were collected for morphometric and histological analyses. The tissues were inflated via the trachea with 4% paraformaldehyde at a constant pressure of 15 cm H_2_O and then fixed for 24 h, paraffin embedded, and sliced into 5-μm-thick sections. Hematoxylin and eosin (H&E) staining was then performed for morphological and histological evaluations under the light microscope. The histological assessment was independently performed by two board-certified pathologists who were blinded to the group allocation.

### Immunofluorescence staining

Tissue sections were dewaxed and dehydrated, followed by antigen retrieval in boiling EDTA antigen-retrieval buffer. The sections were incubated overnight at 4 °C with primary antibodies (α–SMA, 1:500, abcam, Cat. ab5694). After three washes with PBS, the sections were incubated with secondary antibodies (FITC-conjugated goat anti–rabbit IgG, abcam, Cat. ab6717) for 1 h at room temperature. Images were captured at room temperature using a Leica TCS SP5 (Leica Microsystems). Random microscopic fields were selected from each section to calculate the relative fluorescence intensity, and the mean value was determined for each rat.

### Image acquisition and analysis

Image J and Image-Pro Plus 6.0 software were used for image acquisition and analysis to evaluate pulmonary microvessel thickness, radical alveolar count (RAC), mean linear intercept (MLI), and right-heart index (RI), followed by statistical analysis.

### Statistical analysis

All results are presented as the mean ± SEM. After the normality test, all data were in accordance with normal distribution. The normality of data distribution was assessed using the Shapiro–Wilk test before the application of parametric tests. For all data sets, including those with *n* = 4 per group, the Shapiro–Wilk test was used; however, we acknowledge that the power of the test is limited with small sample sizes, and results should be interpreted with caution. Statistical comparisons were performed using two-sample *t*-tests for pairwise comparisons and one-way analysis of variance (ANOVA) for multiple group comparisons. Data analysis was performed using GraphPad Prism version 7 for Windows (GraphPad, San Diego, CA). Statistical significance is denoted as***p < 0.001, **p < *0.01*, and* p < 0.05.

## Results

### Body weight changes and survival rates

**Fig. 2:**
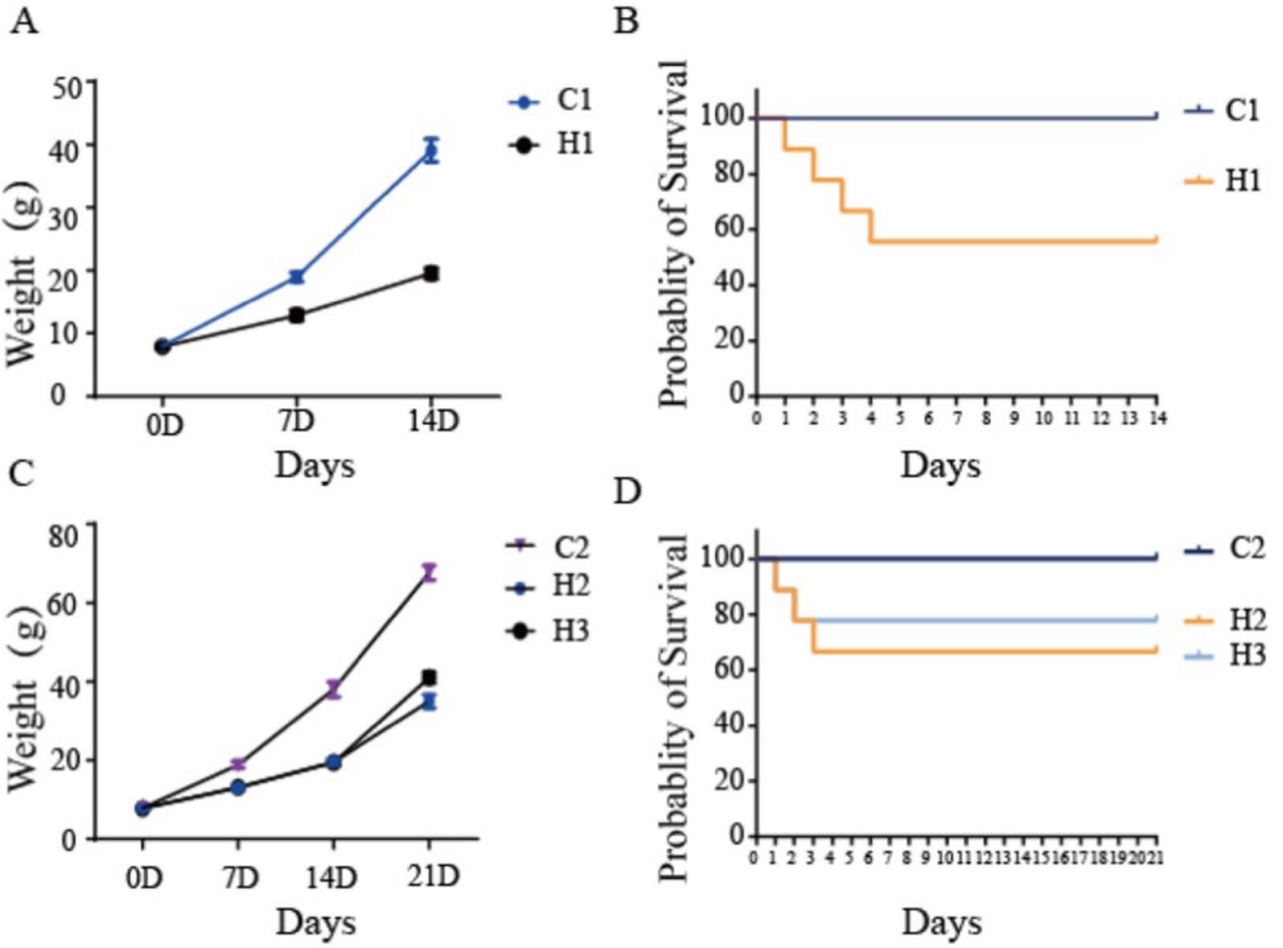
Rat body weight change curves and survival probabilities. C1 represents Control Group 1. C2 represents Control Group 2. H1 represents Experimental Group 1. H2 represents Experimental Group 2. H3 represents Experimental Group 3

Body weight of the rats was measured on days 1, 7, 14, and 21 after application of different modeling methods. The results showed that body weight of the model groups was consistently lower than that of the normal control groups, and the difference became increasingly significant over time (Fig. 2A, C). No death occurred during this period in the control groups, while 3 deaths occurred in the H1 group, 4 in the H2 group, and 3 in the H3 group (Fig. 2B, D). Despite these deaths, each experimental group included at least 4 surviving animals, and all statistical analyses were conducted using data from these survivors only. Although the similar sample size minimized group imbalance, the missing data might have introduced bias or affected the statistical power. Therefore, the conclusions should be interpreted with caution, and this limitation has been acknowledged in the discussion section.

### Hemodynamic assessment of different BPD-PH rat models

TAPSE can effectively reflect right ventricular systolic function and is commonly used to evaluate treatment responses and survival rates. As TAPSE increases with the progression of pregnancy and decreases in infants with BPD-PH, it serves as a reliable indicator of right ventricular dysfunction. Additionally, a decrease in the PAT/PET ratio and an increase in the right-to-left ventricular area (RV/LV) ratio both indicate the presence of pulmonary arterial hypertension.

**Fig. 3:**
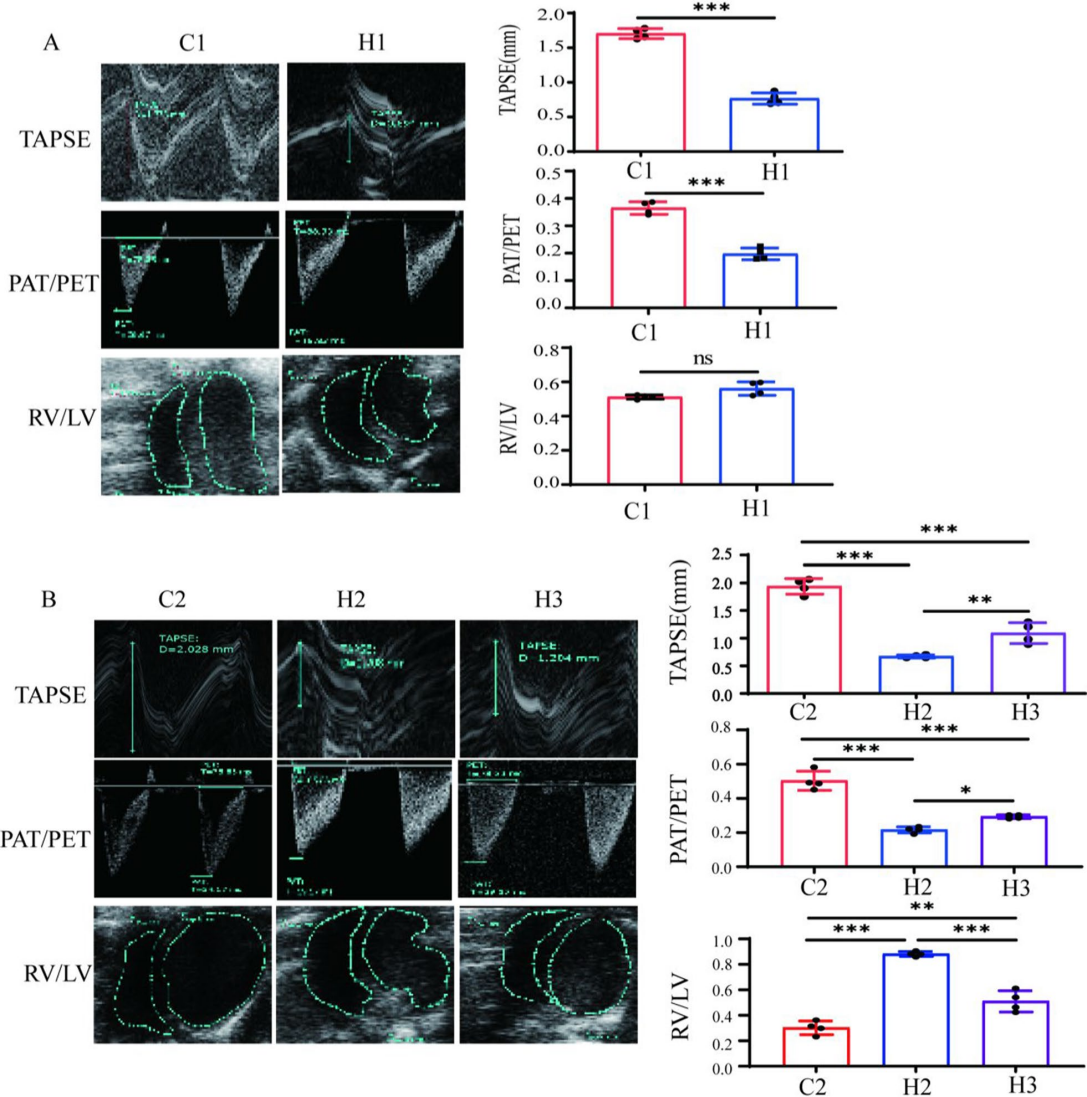
Effects of different modeling methods on ultrasonic hemodynamics of newborn rats. C1 represents Control Group 1. C2 represents Control Group 2. H1 represents Experimental Group 1. H2 represents Experimental Group 2. H3 represents Experimental Group 3. Figures A and B, respectively, show the typical echocardiograms for measuring TAPSE, PAT/PET, right ventricular (RV) and left ventricular (LV) areas of each group (*n* = 4), as well as the statistical results of TAPSE, PAT/PET, and the RV/LV ratio; *P < 0.05, **P < 0.01, ***P < 0.001. *TAPSE* tricuspid annular plane systolic excursion, *PAT* the pulmonary acceleration time, *PET* pulmonary ejection time, *RV* right ventricular area *LV* left ventricular area

After completion of the 2-week modeling of the first-batch rats, both TAPSE and PAT/PET ratios were decreased significantly in the H1 group as compared with those in the C1 control group, though the difference in the RV/LV ratio was not statistically significant between the H1 group and the C1 control group, indicating that the modeling effect of the model groups within two weeks needed to be optimized (Fig. 3A).

After completion of 3-week modeling of the second-batch rats, the TAPSE and PAT/PET ratios in H2 and H3 groups were significantly decreased as compared with those in the control groups, and the RV/LV ratio was increased significantly. The overall performance of the H2 group was better than that of the H3 group, indicating that the 3-week modeling effect was better than the 2-week modeling effect. Even after the hyperoxic injury and subsequent return to normoxia, pulmonary hypertension continued to progress (Fig. 3B).

**Fig. 4:**
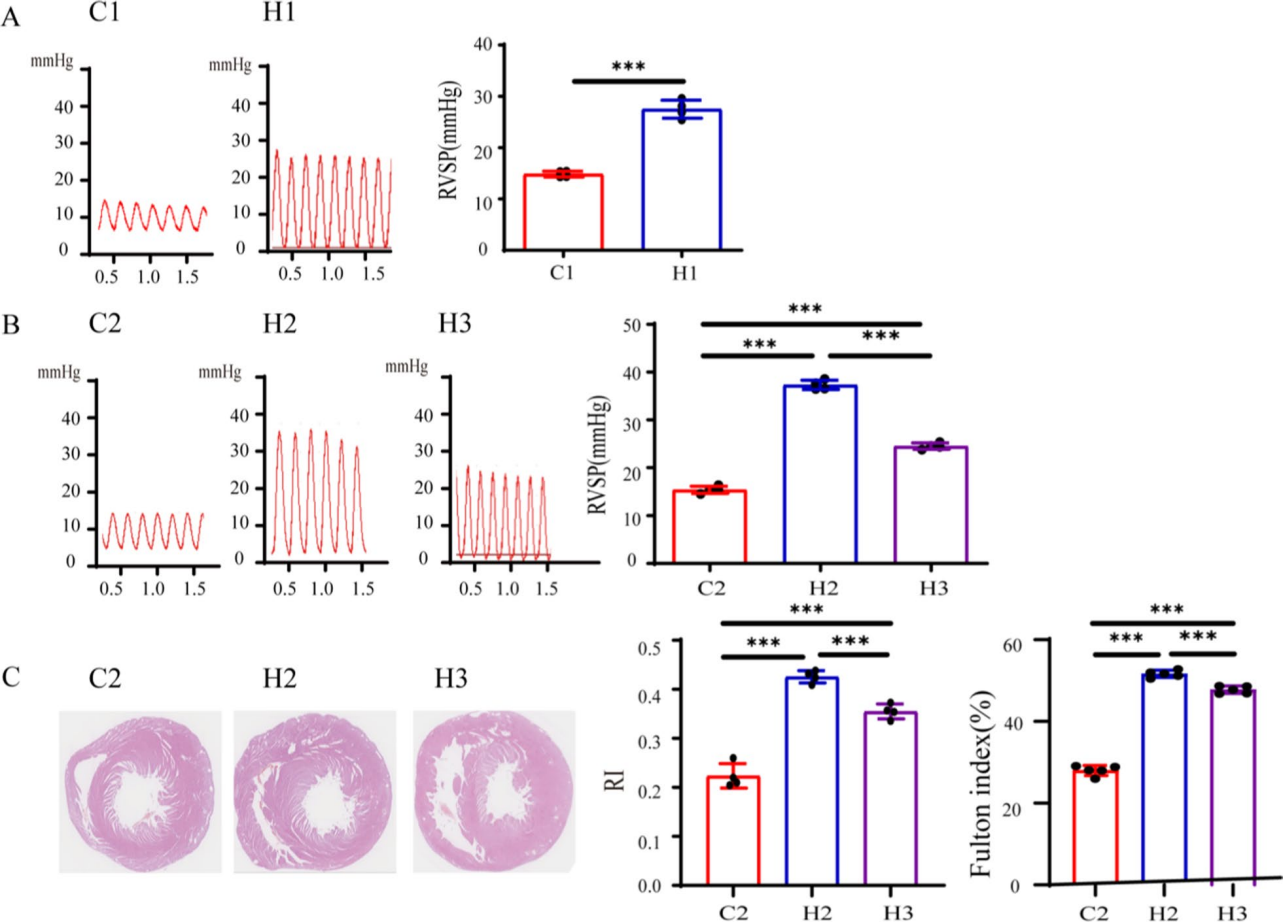
Effects of different modeling methods on RVSP and RI in newborn rats. C1 represents Control Group 1. C2 represents Control Group 2. H1 represents Experimental Group 1. H2 represents Experimental Group 2. H3 represents Experimental Group 3. Figures A and B, respectively, show the RVSP of different groups and the statistical results of RVSP (*n* = 4). Figure C shows the cardiac pathological sections and the statistical results of RI and Fulton index (*n* = 4), *P < 0.05, **P < 0.01, ***P < 0.001. *RVSP* right ventricular systolic pressure, *RI* the right-heart index

After completion of 2-week modeling of the first-batch rats, ultrasonography was performed, and RVSP was measured by catheterization. Heart rate was continuously monitored via electrocardiography throughout the cardiac catheterization procedure and maintained within 300–450 bpm to ensure accurate and comparable cardiac function measurements in 14 or 21-day-old rats. The results showed that RVSP in the H1 group was significantly higher than that in the control groups (Fig. 4A). After completion of 3-week modeling of the second-batch rats, RVSP was measured by cardiac catheterization, and the result showed that RVSP in both the H2 and H3 groups was significantly higher than that in the control groups, and RVSP in the H2 group was also significantly higher than that in the H3 group (Fig. 4B), indicating that all model groups were successfully modeled, and the H2 group exhibited the effect.

Subsequently, the second-batch modeled rats were sacrificed; the lung tissues and hearts were isolated; the bronchi and surrounding connective tissues were excised; the hearts were H&E stained for calculating the RI and Fulton index (%) (Fig. 4C). The results showed that the right ventricles in H2 and H3 groups were significantly thicker than those in the normal control group, and the thickening in the H2 group was the most obvious.

### Lung tissue damage and pulmonary vascular remodeling

**Fig. 5:**
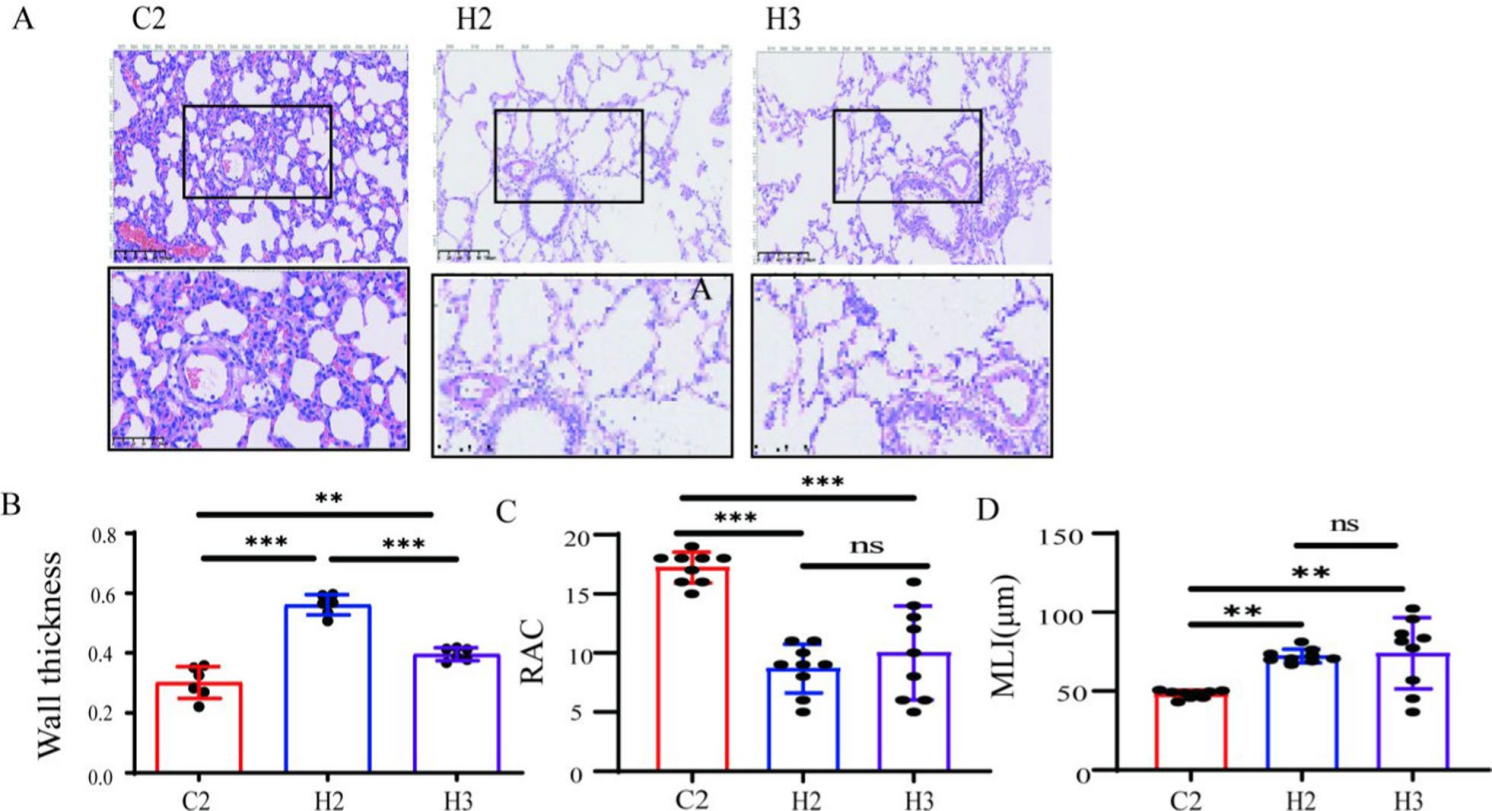
Effects of different modeling methods on BPD–PH lung parenchymal injury and pulmonary vascular remodeling. C2 represents Control Group 2. H2 represents Experimental Group 2. H3 represents Experimental Group 3. A Shows the HE-staining of lung tissues (20X), with an enlarged view of the pulmonary vascular region in the box (40X); B illustrates the statistical results of pulmonary vascular thickness (*n* = 4); C presents the statistical results of RAC (*n* = 4); D shows the statistical results of MLI (*n* = 4); *P < 0.05, **P < 0.01, ***P < 0.001. *RAC* radical alveolar count, *MLI* mean linear intercept

To assess lung tissue damage and progression, MLI, RAC, and pulmonary artery wall thickness were estimated by lung histology. Alveolar development was evaluated by MLI and RAC, and pulmonary vascular remodeling was evaluated by pulmonary artery wall thickness. Compared with the control groups, MLI was increased and RAC was decreased in all model groups, indicating that all these modeling methods were able to inhibit alveolar development (Fig. 5).

The H&E staining results of the control groups showed that the alveoli were relatively uniform in size, with intact and appropriately thick septa, and a reasonable density of capillary distribution. In contrast, the alveolar volume in the model groups increased significantly, the number of blood vessels decreased, the structure was simplified, the normal morphology was lost, and the alveoli fused into large bullae. The secondary septa decreased, accompanied by a significant reduction in blood vessels, and the pulmonary arterioles showed muscularization and thickening of the vascular walls. After subsequent normoxic exposure, the alveolar septa in some areas became significantly thickened, while obvious large bullae appeared in other areas, resulting in uneven alveolar sizes. These findings suggest that hyperoxia had a destructive effect on the lung tissues and pulmonary vessels. However, in rats subjected to subsequent normoxic exposure, the lung tissues remodeled again, ultimately leading to varying degrees of pulmonary vascular remodeling and right ventricular hypertrophy.

### High expression of α-SMA in the pulmonary artery smooth muscle of model rats

**Fig. 6:**
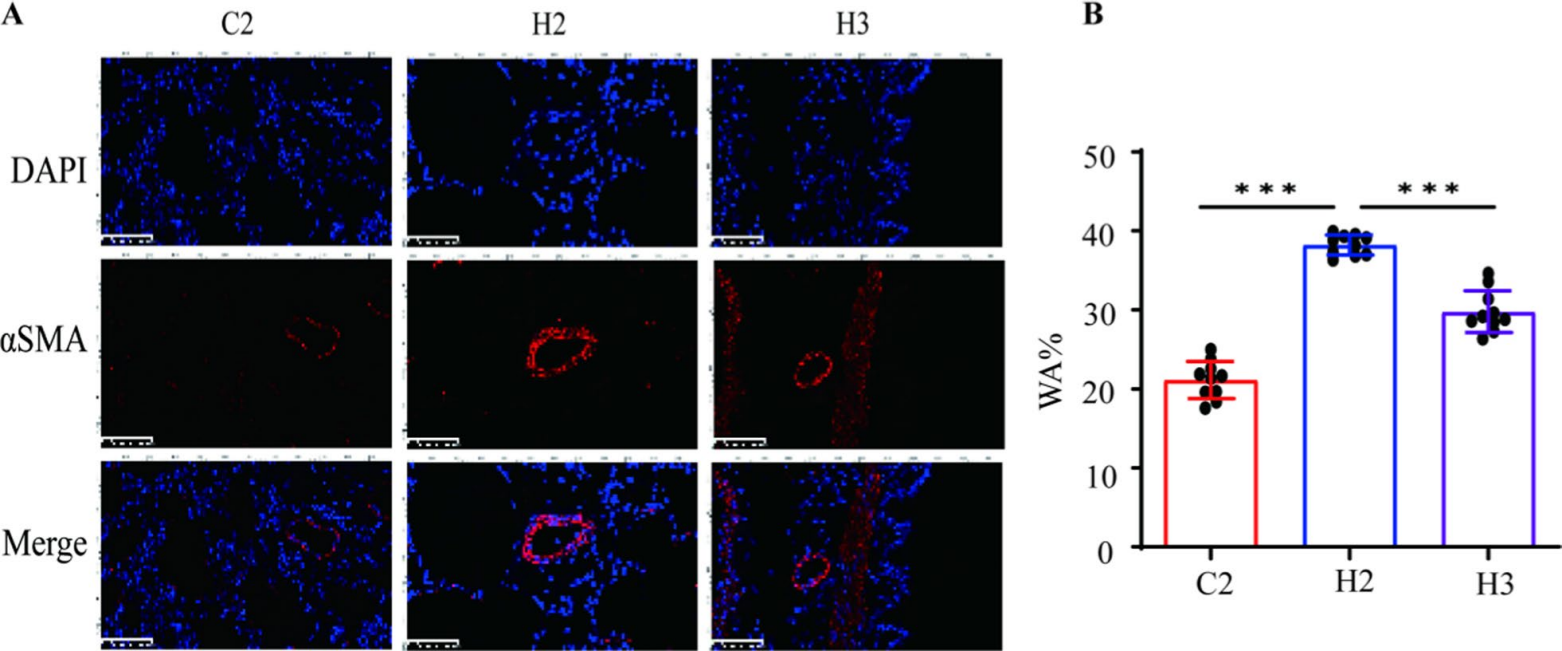
Effects of different modeling methods on BPD–PH pulmonary vascular remodeling. C2 represents Control Group 2. H2 represents Experimental Group 2. H3 represents Experimental Group 3. The more red observed in the vascular wall, the more pronounced the vascular remodeling; A presents Lung tissue α-SMA immunofluorescence staining; B shows the statistical results of WA% (*n* = 4); *P < 0.05, **P < 0.01, ***P < 0.001. *α-SMA* α–Smooth muscle actin, *WA%* wall area percentage

Alpha-smooth muscle actin (α-SMA) is a protein marker commonly used to identify smooth muscle cells and myofibroblasts. The density of α-SMA-positive cells can serve as an indicator of tissue repair, fibrosis, and disease progression. Immunofluorescence staining of α-SMA was performed on paraffin-embedded sections of the lung tissues to reflect pulmonary vascular remodeling in each group. Compared with the control groups, the pulmonary vessels in all model groups showed varying degrees of thickening, reflecting different levels of pulmonary vascular remodeling in the model groups. Notably, the pulmonary vascular staining in the H2 group was the most pronounced, suggesting that the pulmonary vascular remodeling in the H2 group was more significant than that in the other model groups (Fig. 6).

## Discussion

In this study, we successfully established a sequential hyperoxia–hypoxia exposure model (H2 group), which dynamically replicated the clinical progression of BPD-PH with two-stage pathological features. Early hyperoxia (75% O_2_ for 2 weeks) induced alveolar simplification (a 50% increase in MLI), and subsequent hypoxia (10% O_2_ exposure for a week) drove pulmonary vascular remodeling. These findings are consistent with the clinical course of BPD-PH in the patient subgroup who experience a sequential hyperoxia-to-hypoxia exposure, which involves initial alveolar injury followed by hypoxia-mediated PH [21]. The model also well replicated the right-ventricular dysfunction features of BPD-PH. In the H2 group, RVSP (37.33 ± 0.99 mmHg) and the RV/LV ratio (0.88 ± 0.02) were significantly elevated, exceeding the values in the single hyperoxia model (H1 group: RVSP = 27.51 ± 1.75 mmHg), reflecting the right heart failure phenotype in human BPD-PH infants [22].

Traditional single hyperoxia models can effectively simulate the arrest of alveolar development, as demonstrated by our H1 group, but they cannot fully reproduce hypoxia-driven vascular remodeling (Figs. 5, 6) and lack the dynamic changes of disease progression [23]. Hypoxia- or inflammation-based models, such as the lipopolysaccharide (LPS)-hyperoxia model, can induce inflammation but cannot distinguish the roles of hyperoxia and hypoxia at different stages and have a high mortality rate (> 40%) [24, 25]. The sequential hyperoxia–hypoxia exposure model, as we developed in the H2 group, incorporated time-dependent oxygen exposure and therefore can simulate the clinical situation more realistically, thus filling the "dynamic mechanism gap" emphasized by Hansmann et al. [26] and providing a more clinically relevant alternative to the two-factor model (hyperoxia + LPS) by Garrick et al. [9].

Clinically, the occurrence of BPD-PH is closely related to alveolar simplification and blockade of microvascular angiogenesis caused by early hyperoxia, as well as pulmonary vascular remodeling induced by subsequent relative hypoxia. The sequential hyperoxia–hypoxia exposure model established in this study (H2 group) effectively replicates this pathophysiological process. During the hyperoxia stage, long-term exposure to 75% O_2_ impairs the differentiation of alveolar epithelial cells (such as the blocked transformation of AT2 to AT1) through oxidative stress (such as the down-regulation of superoxide dismutase 2, SOD2), resulting in alveolar simplification [27]. At the same time, hyperoxia-induced apoptosis of microvascular endothelial cells makes the lungs more sensitive to hypoxia [28]. During the subsequent hypoxia stage, chronic hypoxia activates the proliferation of pulmonary artery smooth muscle cells (PASMCs) through the HIF-1α signaling pathway [29] and increases the secretion of endothelin 1 (ET-1) [30], exacerbating pulmonary vascular resistance. This is consistent with the enhanced hypoxic pulmonary vasoreactivity observed in BPD-PH patients [31].

The successful establishment of the sequential hyperoxia–hypoxia exposure model in this study provides a valuable experimental platform for investigating the mechanisms of oxygen-induced lung injury and the potential impact of sequential oxygen exposures. While our findings may offer insights to guide future research on oxygen therapy strategies in preterm infants, we acknowledge that direct clinical translation is limited by interspecies differences in oxygen response and the need for further validation in large animal models and clinical settings. Therefore, our results should be interpreted as providing preclinical evidence to inform, rather than to directly dictate, clinical protocols [32].

In addition, this model also brings inspiration for stage-specific treatment of BPD-PH. The model can evaluate stage-specific interventions, such as the use of antioxidants during the hyperoxia period and ET-1 receptor antagonists during the hypoxia period, addressing the limitations of the current “one-size-fits-all” treatment methods [33].

Although we have established a BPD-PH model that well replicates clinical features through sequential hyperoxia–hypoxia exposure, the limitations of this model should be recognized. First, the clinical pathological process of BPD-PH is very complex, and oxygen therapy is only one of the factors. The failure to include clinical confounding factors (such as mechanical ventilation and infection) in this model may underestimate the interactions of multiple factors [34]. Secondly, although each group maintained four survivors for analysis, animal deaths could potentially introduce bias or reduce statistical power, especially if related to experimental treatment. This possibility should be considered in the interpretation of our results. In addition, there are cross-species differences between rodents and humans, with obvious differences in the post-natal alveolarization timeline, which need to be verified in large animal models (such as preterm lambs) [35]. Finally, the mechanism of the formation of this model still needs further exploration. Although an increase in α-SMA was observed in this study, the regulatory network of smooth muscle cell phenotypic transformation (such as MYH11/SM22α) has not been explored in depth [36].

## Conclusion

This study pioneered a sequential hyperoxia–hypoxia model that truly replicated the dynamic progression of BPD-PH from alveolar injury to pulmonary hypertension. By overcoming the limitations of the single exposure mode, this model provides a powerful tool for studying stage-specific mechanisms and developing precise treatments. In the future, the integration of multi-omics technologies and translational research will accelerate breakthroughs in the treatment of BPD-PH.

## Data Availability

Data will be made available upon reasonable request.
